# Crystal structure of *catena*-poly[1,3-di­benzyl­benzimidazolium [[chlorido­mercurate(II)]-di-μ-chlorido]]

**DOI:** 10.1107/S2056989015023427

**Published:** 2015-12-12

**Authors:** Mehdi Bouchouit, Abdelmalek Bouraiou, Sofiane Bouacida, Hocine Merazig, Ali Belfaitah, Mebarek Bahnous

**Affiliations:** a Unité de Recherche de Chimie de l’Environnement et Moléculaire Structurale, CHEMS, Université Frères Montouri Constantine, 25000, Algeria; b Département Sciences de la Matière, Faculté des Sciences Exactes et Sciences de la Nature et de la Vie, Université Oum El Bouaghi, Algeria; c Laboratoire des Produits Naturels d’Origine Végétale et de Synthèse Organique, PHYSYNOR; Université Frères Montouri Constantine, 25000 Constantine, Algeria; d Département de Chimie, Université frères Montouri Constantine, 25000 , Algeria

**Keywords:** crystal structure, benzimidazole derivative, tri­chlorido­mercurate

## Abstract

The asymmetric unit of the polymeric title compound, {(C_21_H_19_N_2_)[HgCl_3_]}_*n*_, comprises one-half of the cationic mol­ecule, the other half being generated by application of twofold rotation symmetry, one Hg and two Cl atoms. The Hg^II^ atom, lying on a twofold rotation axis, exhibits a distorted triangular coordination environment and is surrounded by three Cl atoms with Hg—Cl distances in the range 2.359 (2)–2.4754 (13) Å. Two additional longer distances [Hg⋯Cl = 3.104 (14) Å] lead to the formation of polymeric [HgCl_1/1_Cl_4/2_]^−^ chains extending along [001]. The crystal packing can be described by cationic layers alternating parallel to (-110) with the anionic chains located between the layers. The packing is consolidated by π–π stacking inter­actions between the benzene rings of the central benzimidazole entities, with centroid-to-centroid distances of 3.643 (3) Å.

## Related literature   

Benzimidazoles and their derivatives show anti-oxidant (Kuş *et al.*, 2004 ▸), anti­fungal (Preston, 1974 ▸) and anthelminthic (Hazelton *et al.*, 1995 ▸) properties and have applications in pharmacy and agriculture (Malek *et al.*, 2006 ▸). They can also be used as ep­oxy resin curing agents, catalysts, metallic surface treatment agents (Li *et al.*, 2003 ▸; Abboud *et al.*, 2006 ▸) or as ionic liquids (Li *et al.*, 2011 ▸; Chen *et al.*, 2008 ▸). For the importance of transition metals ions in biological processes, see: Kaim & Schwederski (1994 ▸). For bond lengths of delocalized systems, see: Ennajih *et al.* (2009 ▸).
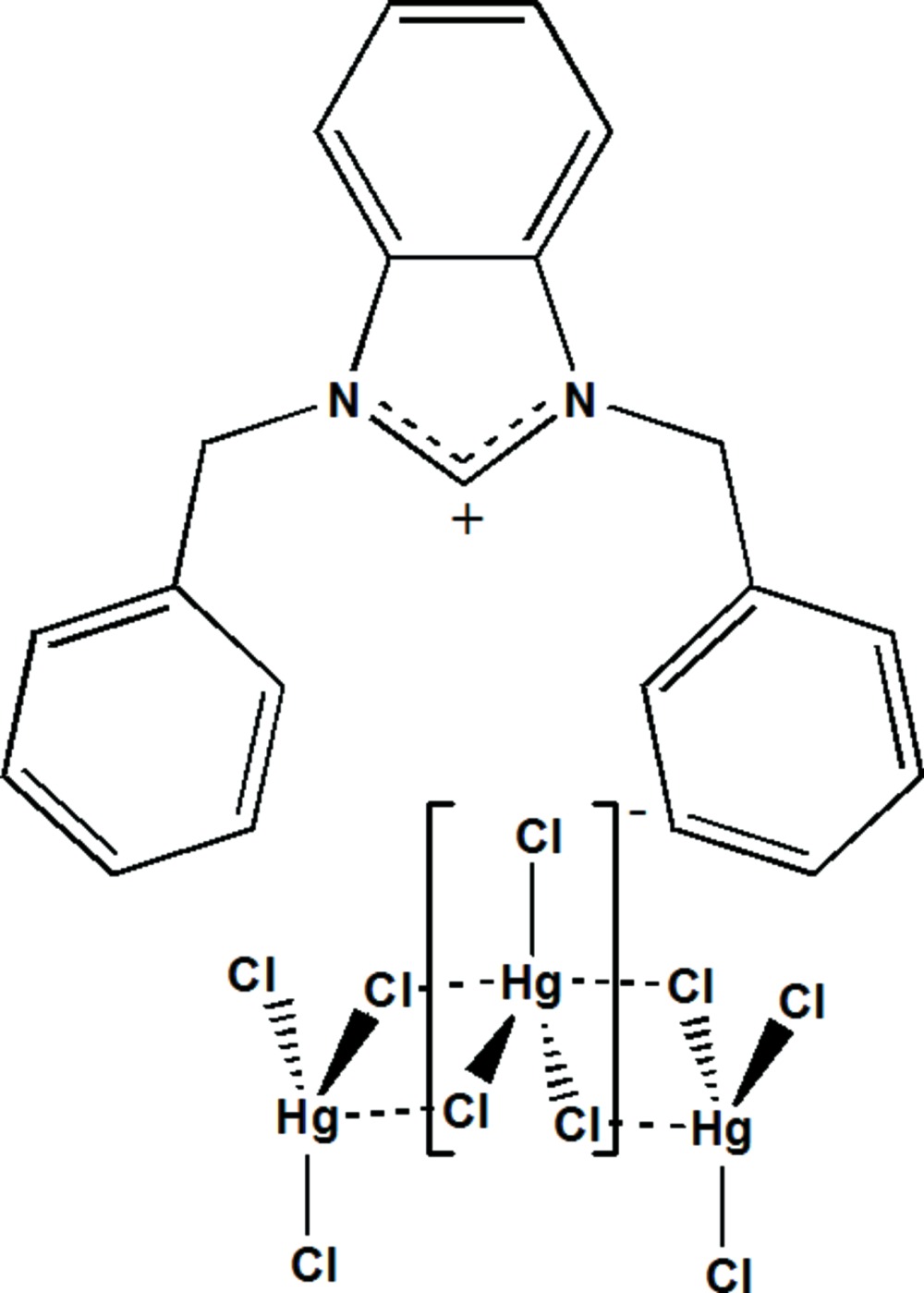


## Experimental   

### Crystal data  

(C_21_H_19_N_2_)[HgCl_3_]
*M* *_r_* = 606.32
Monoclinic, 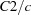

*a* = 20.3669 (11) Å
*b* = 14.8837 (7) Å
*c* = 7.2154 (4) Å
β = 104.372 (2)°
*V* = 2118.79 (19) Å^3^
*Z* = 4
Mo *K*α radiation
μ = 7.65 mm^−1^
*T* = 295 K
0.19 × 0.11 × 0.05 mm

### Data collection  

Bruker APEXII CCD diffractometer
Absorption correction: multi-scan (*SADABS*; Bruker, 2011 ▸) *T*_min_ = 0.646, *T*_max_ = 0.746
8306 measured reflections
2401 independent reflections
1571 reflections with *I* > 2σ(*I*)
*R*_int_ = 0.042

### Refinement  

*R*[*F* ^2^ > 2σ(*F* ^2^)] = 0.038
*wR*(*F* ^2^) = 0.085
*S* = 1.00
2401 reflections
124 parameters
H-atom parameters constrained
Δρ_max_ = 2.02 e Å^−3^
Δρ_min_ = −0.41 e Å^−3^

### 

Data collection: *APEX2* (Bruker, 2011 ▸); cell refinement: *SAINT* (Bruker, 2011 ▸); data reduction: *SAINT*; program(s) used to solve structure: *SIR2002* (Burla *et al.*, 2005 ▸); program(s) used to refine structure: *SHELXL97* (Sheldrick, 2008 ▸); molecular graphics: *ORTEP-3 for Windows* (Farrugia, 2012 ▸) and *DIAMOND* (Brandenburg & Berndt, 2001 ▸); software used to prepare material for publication: *WinGX* (Farrugia, 2012 ▸).

## Supplementary Material

Crystal structure: contains datablock(s) I. DOI: 10.1107/S2056989015023427/wm5248sup1.cif

Structure factors: contains datablock(s) I. DOI: 10.1107/S2056989015023427/wm5248Isup2.hkl

**Fig. 1.**
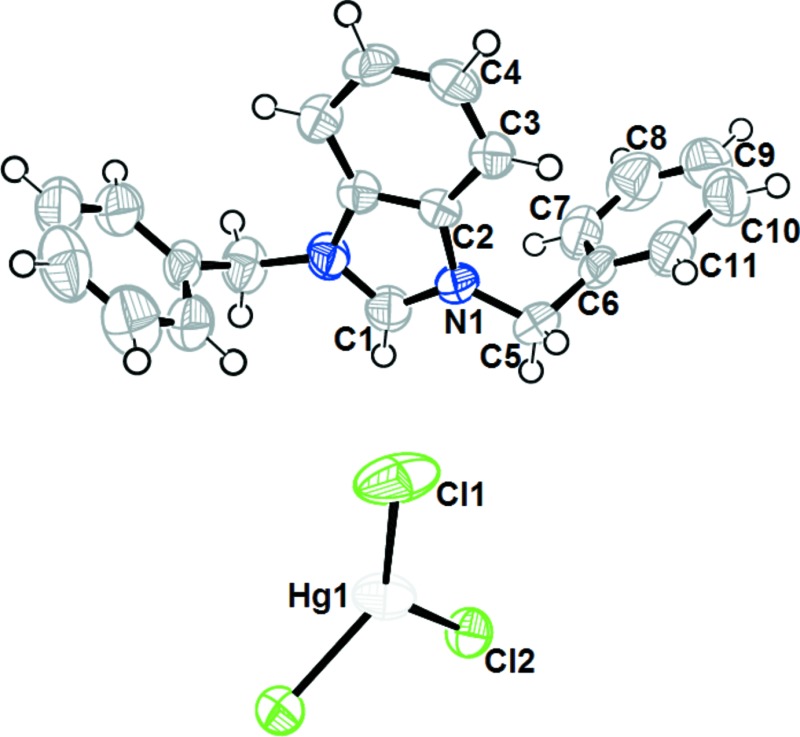
The molecular structures of the entities in the title compound. Displacement ellipsoids are drawn at the 50% probability level; H atoms are represented as small spheres of arbitrary radius. Non-labelled atoms are generated by symmetry code 2 − x, y, 3/2 − z for the cation and by symmetry code 2 − x, y, 1/2 − z for the anion.

Click here for additional data file.x y z x y z . DOI: 10.1107/S2056989015023427/wm5248fig1.tif
The mol­ecular structures of the entities in the title compound. Displacement ellipsoids are drawn at the 50% probability level; H atoms are represented as small spheres of arbitrary radius. Non-labelled atoms are generated by symmetry code 2 − *x*, *y*, 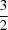 − *z* for the cation and by symmetry code 2 − *x*, *y*, 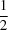 − *z* for the anion.

**Fig. 2.**
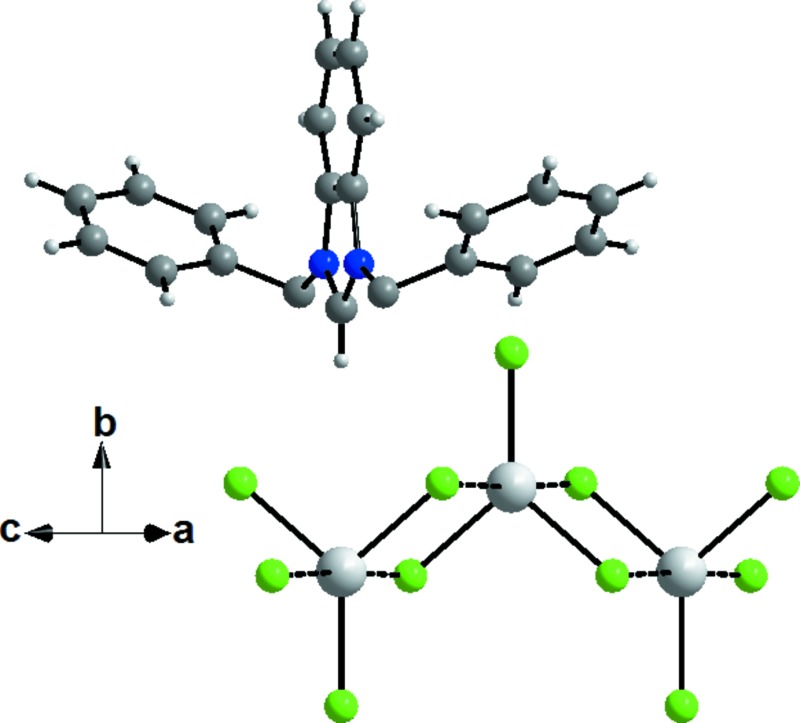
The polymeric anionic [HgCl1/1Cl4/2]− chain defined by long Hg—Cl distances (in dashed lines).

1/1 4/2 − . DOI: 10.1107/S2056989015023427/wm5248fig2.tif
The polymeric anionic [HgCl_1/1_Cl_4/2_]^−^ chain defined by long Hg—Cl distances (in dashed lines).

**Fig. 3.**
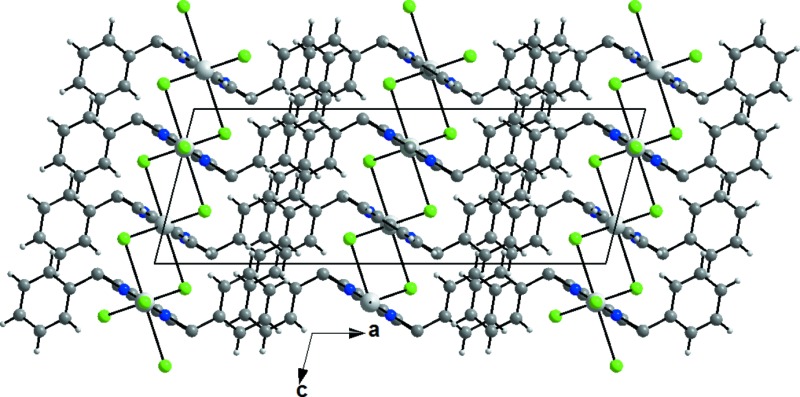
The crystal packing of the title compound viewed down [010] showing alternating layers of cations and anions.

. DOI: 10.1107/S2056989015023427/wm5248fig3.tif
The crystal packing of the title compound viewed down [010] showing alternating layers of cations and anions.

**Fig. 4.**
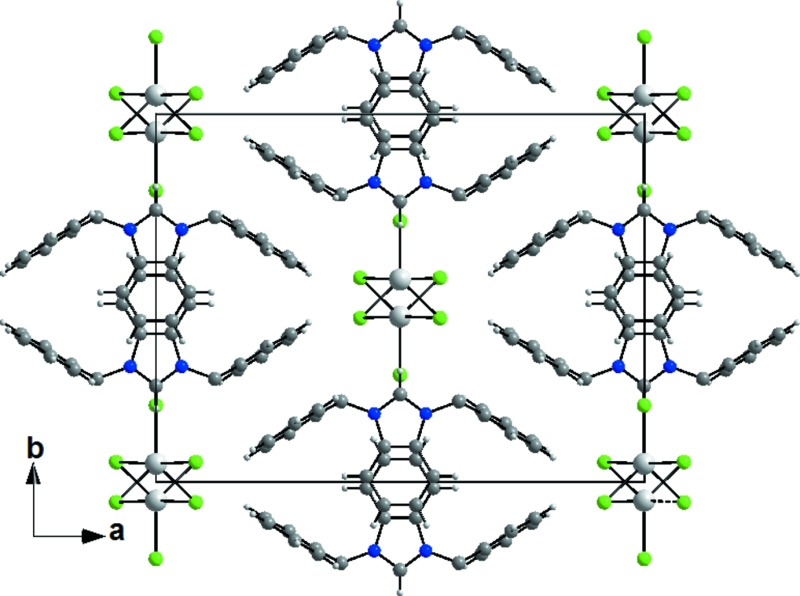
The crystal packing of the title compound viewed down [001] (chain direction).

. DOI: 10.1107/S2056989015023427/wm5248fig4.tif
The crystal packing of the title compound viewed down [001] (chain direction).

CCDC reference: 1440716

Additional supporting information:  crystallographic information; 3D view; checkCIF report

## supplementary crystallographic information

### S1. Experimental

1,3-Dibenzylbenzimidazolium trichloridomercurate(II) was synthesized by reaction of 1 mmol of 1,3-dibenzylbenzimidazolium chloride with 1 mmol of mercury(II) chloride in methanol at room temperature. The solid obtained was recrystallized in methanol to yield yellow crystals of the title compound suitable for X-ray diffraction.

### S2. Refinement

H atoms were localized from difference maps but were modelled in calculated positions and treated as riding on their parent atom with C—H = 0.93 Å (aromatic), C—H = 0.97 Å (methylene) with *U*_iso_(H) = 1.2*U*_eq_(C_aromatic_ or C_methylene_).

### Crystal data

**Table:**

(C_21_H_19_N_2_)[HgCl_3_]	*F*(000) = 1160
*M**_r_* = 606.32	*D*_x_ = 1.901 Mg m^−^^3^
Monoclinic, *C*2/*c*	Mo *K*α radiation, λ = 0.71073 Å
Hall symbol: -C 2yc	Cell parameters from 2352 reflections
*a* = 20.3669 (11) Å	θ = 2.7–22.8°
*b* = 14.8837 (7) Å	µ = 7.65 mm^−^^1^
*c* = 7.2154 (4) Å	*T* = 295 K
β = 104.372 (2)°	Prism, yellow
*V* = 2118.79 (19) Å^3^	0.19 × 0.11 × 0.05 mm
*Z* = 4	

### Data collection

**Table:**

Bruker APEXII CCD diffractometer	2401 independent reflections
Radiation source: Enraf Nonius FR590	1571 reflections with *I* > 2σ(*I*)
Graphite monochromator	*R*_int_ = 0.042
CCD rotation images, thick slices scans	θ_max_ = 27.5°, θ_min_ = 2.7°
Absorption correction: multi-scan (*SADABS*; Bruker, 2011)	*h* = −26→26
*T*_min_ = 0.646, *T*_max_ = 0.746	*k* = −18→13
8306 measured reflections	*l* = −9→9

### Refinement

**Table:**

Refinement on *F*^2^	Primary atom site location: structure-invariant direct methods
Least-squares matrix: full	Secondary atom site location: difference Fourier map
*R*[*F*^2^ > 2σ(*F*^2^)] = 0.038	Hydrogen site location: inferred from neighbouring sites
*wR*(*F*^2^) = 0.085	H-atom parameters constrained
*S* = 1.00	*w* = 1/[σ^2^(*F*_o_^2^) + (0.0419*P*)^2^] where *P* = (*F*_o_^2^ + 2*F*_c_^2^)/3
2401 reflections	(Δ/σ)_max_ = 0.002
124 parameters	Δρ_max_ = 2.02 e Å^−^^3^
0 restraints	Δρ_min_ = −0.41 e Å^−^^3^

### Special details

**Table:**

Geometry. All e.s.d.'s (except the e.s.d. in the dihedral angle between two l.s. planes) are estimated using the full covariance matrix. The cell e.s.d.'s are taken into account individually in the estimation of e.s.d.'s in distances, angles and torsion angles; correlations between e.s.d.'s in cell parameters are only used when they are defined by crystal symmetry. An approximate (isotropic) treatment of cell e.s.d.'s is used for estimating e.s.d.'s involving l.s. planes.
Refinement. Refinement of *F*^2^ against ALL reflections. The weighted *R*-factor *wR* and goodness of fit *S* are based on *F*^2^, conventional *R*-factors *R* are based on *F*, with *F* set to zero for negative *F*^2^. The threshold expression of *F*^2^ > σ(*F*^2^) is used only for calculating *R*-factors(gt) *etc*. and is not relevant to the choice of reflections for refinement. *R*-factors based on *F*^2^ are statistically about twice as large as those based on *F*, and *R*- factors based on ALL data will be even larger.

### Fractional atomic coordinates and isotropic or equivalent isotropic displacement parameters (Å^2^)

**Table:**

	*x*	*y*	*z*	*U*_iso_*/*U*_eq_	
Hg1	1	0.050020 (19)	0.25	0.05935 (16)	
Cl2	0.91850 (7)	−0.05454 (8)	0.33958 (18)	0.0473 (3)	
Cl1	1	0.20850 (14)	0.25	0.0888 (9)	
N1	0.9472 (2)	0.3140 (3)	0.6672 (6)	0.0415 (10)	
C4	1	0.2622 (5)	0.75	0.0484 (19)	
H4	1	0.1998	0.75	0.058*	
C6	0.8265 (3)	0.3186 (3)	0.6596 (8)	0.0446 (13)	
C3	0.9676 (2)	0.4034 (3)	0.6988 (6)	0.0348 (11)	
C1	0.9672 (3)	0.5616 (3)	0.6966 (8)	0.0463 (14)	
H1	0.9458	0.6165	0.6613	0.056*	
C2	0.9314 (3)	0.4838 (4)	0.6393 (7)	0.0418 (12)	
H2	0.8868	0.4841	0.5667	0.05*	
C5	0.8795 (3)	0.2814 (4)	0.5694 (8)	0.0524 (14)	
H5A	0.8789	0.2163	0.5749	0.063*	
H5B	0.8694	0.299	0.4359	0.063*	
C7	0.8324 (3)	0.3053 (4)	0.8527 (9)	0.0569 (15)	
H7	0.8683	0.2721	0.9258	0.068*	
C11	0.7722 (3)	0.3668 (4)	0.5561 (9)	0.0574 (15)	
H11	0.768	0.3764	0.4263	0.069*	
C8	0.7847 (4)	0.3418 (5)	0.9352 (9)	0.0751 (19)	
H8	0.7893	0.3342	1.0658	0.09*	
C9	0.7303 (3)	0.3891 (5)	0.8308 (12)	0.075 (2)	
H9	0.6982	0.4127	0.8894	0.091*	
C10	0.7238 (3)	0.4013 (4)	0.6396 (12)	0.0695 (18)	
H10	0.6869	0.4327	0.5663	0.083*	

### Atomic displacement parameters (Å^2^)

**Table:**

	*U*^11^	*U*^22^	*U*^33^	*U*^12^	*U*^13^	*U*^23^
Hg1	0.0830 (3)	0.02880 (19)	0.0678 (2)	0	0.02171 (18)	0
Cl2	0.0457 (8)	0.0495 (8)	0.0472 (7)	−0.0016 (6)	0.0120 (6)	0.0026 (6)
Cl1	0.146 (3)	0.0298 (12)	0.0719 (15)	0	−0.0079 (15)	0
N1	0.037 (3)	0.031 (2)	0.053 (3)	−0.0031 (19)	0.006 (2)	−0.0073 (19)
C4	0.043 (5)	0.033 (4)	0.068 (5)	0	0.010 (4)	0
C6	0.031 (3)	0.032 (3)	0.064 (4)	−0.005 (2)	−0.002 (3)	−0.008 (2)
C3	0.037 (3)	0.033 (3)	0.036 (3)	0.003 (2)	0.012 (2)	−0.003 (2)
C1	0.055 (3)	0.029 (3)	0.056 (3)	0.011 (2)	0.014 (3)	0.006 (2)
C2	0.039 (3)	0.042 (3)	0.042 (3)	0.003 (2)	0.006 (2)	−0.003 (2)
C5	0.044 (3)	0.048 (3)	0.059 (3)	−0.005 (3)	0.001 (3)	−0.018 (3)
C7	0.042 (4)	0.060 (4)	0.063 (4)	−0.004 (3)	0.001 (3)	0.003 (3)
C11	0.048 (4)	0.056 (4)	0.062 (4)	−0.007 (3)	0.001 (3)	0.002 (3)
C8	0.068 (5)	0.098 (6)	0.062 (4)	−0.013 (4)	0.020 (4)	−0.012 (4)
C9	0.052 (4)	0.075 (5)	0.106 (6)	−0.007 (4)	0.033 (4)	−0.027 (4)
C10	0.043 (4)	0.053 (4)	0.109 (6)	0.000 (3)	0.012 (4)	−0.001 (4)

### Geometric parameters (Å, º)

**Table:**

Hg1—Cl1	2.359 (2)	C1—C2	1.376 (7)
Hg1—Cl2^i^	2.4754 (13)	C1—H1	0.93
Hg1—Cl2	2.4754 (13)	C2—H2	0.93
N1—C4	1.336 (6)	C5—H5A	0.97
N1—C3	1.397 (6)	C5—H5B	0.97
N1—C5	1.466 (6)	C7—C8	1.372 (9)
C4—N1^ii^	1.336 (6)	C7—H7	0.93
C4—H4	0.93	C11—C10	1.375 (9)
C6—C11	1.374 (7)	C11—H11	0.93
C6—C7	1.383 (8)	C8—C9	1.370 (10)
C6—C5	1.499 (7)	C8—H8	0.93
C3—C3^ii^	1.344 (9)	C9—C10	1.365 (10)
C3—C2	1.414 (7)	C9—H9	0.93
C1—C1^ii^	1.367 (11)	C10—H10	0.93
			
Cl1—Hg1—Cl2^i^	128.95 (3)	N1—C5—C6	111.2 (4)
Cl1—Hg1—Cl2	128.95 (3)	N1—C5—H5A	109.4
Cl2^i^—Hg1—Cl2	102.09 (6)	C6—C5—H5A	109.4
C4—N1—C3	107.6 (4)	N1—C5—H5B	109.4
C4—N1—C5	125.5 (4)	C6—C5—H5B	109.4
C3—N1—C5	126.8 (4)	H5A—C5—H5B	108
N1^ii^—C4—N1	109.7 (6)	C8—C7—C6	119.1 (6)
N1^ii^—C4—H4	125.2	C8—C7—H7	120.4
N1—C4—H4	125.2	C6—C7—H7	120.4
C11—C6—C7	118.7 (6)	C6—C11—C10	121.7 (6)
C11—C6—C5	121.8 (5)	C6—C11—H11	119.2
C7—C6—C5	119.5 (5)	C10—C11—H11	119.2
C3^ii^—C3—N1	107.5 (3)	C9—C8—C7	121.9 (6)
C3^ii^—C3—C2	122.2 (3)	C9—C8—H8	119
N1—C3—C2	130.2 (4)	C7—C8—H8	119
C1^ii^—C1—C2	122.7 (3)	C10—C9—C8	119.1 (6)
C1^ii^—C1—H1	118.7	C10—C9—H9	120.4
C2—C1—H1	118.7	C8—C9—H9	120.4
C1—C2—C3	115.1 (5)	C9—C10—C11	119.5 (6)
C1—C2—H2	122.5	C9—C10—H10	120.2
C3—C2—H2	122.5	C11—C10—H10	120.2
			
C3—N1—C4—N1^ii^	0.1 (2)	C11—C6—C5—N1	123.0 (5)
C5—N1—C4—N1^ii^	−177.2 (5)	C7—C6—C5—N1	−56.1 (7)
C4—N1—C3—C3^ii^	−0.2 (6)	C11—C6—C7—C8	−1.0 (8)
C5—N1—C3—C3^ii^	177.1 (5)	C5—C6—C7—C8	178.1 (5)
C4—N1—C3—C2	179.0 (4)	C7—C6—C11—C10	−0.5 (8)
C5—N1—C3—C2	−3.7 (8)	C5—C6—C11—C10	−179.6 (5)
C1^ii^—C1—C2—C3	0.0 (9)	C6—C7—C8—C9	1.7 (10)
C3^ii^—C3—C2—C1	−1.1 (8)	C7—C8—C9—C10	−0.8 (10)
N1—C3—C2—C1	179.8 (5)	C8—C9—C10—C11	−0.7 (10)
C4—N1—C5—C6	121.4 (5)	C6—C11—C10—C9	1.3 (9)
C3—N1—C5—C6	−55.4 (7)		

Symmetry codes: (i) −*x*+2, *y*, −*z*+1/2; (ii) −*x*+2, *y*, −*z*+3/2.

## References

[bb1] Abboud, Y., Abourriche, A., Saffaj, T., Berrada, M., Charrouf, M., Bennamara, A., Cherqaoui, A. & Takky, D. (2006). *Appl. Surf. Sci.* **252**, 8178–8184.

[bb2] Brandenburg, K. & Berndt, M. (2001). *DIAMOND*. Crystal Impact, Bonn, Germany.

[bb3] Bruker (2011). *APEX2*, *SAINT* and *SADABS*. Bruker AXS Inc., Madison, Wisconsin, USA.

[bb4] Burla, M. C., Caliandro, R., Camalli, M., Carrozzini, B., Cascarano, G. L., De Caro, L., Giacovazzo, C., Polidori, G. & Spagna, R. (2005). *J. Appl. Cryst.* **38**, 381–388.

[bb5] Chen, S.-H., Zhao, Q. & Xu, X.-W. (2008). *J. Chem. Sci.* **120**, 481–483.

[bb6] Ennajih, H., Bouhfid, R., Zouihri, H., Essassi, E. M. & Ng, S. W. (2009). *Acta Cryst.* E**65**, o2321. 10.1107/S1600536809034175 PMC2970382 21577792

[bb7] Farrugia, L. J. (2012). *J. Appl. Cryst.* **45**, 849–854.

[bb8] Hazelton, J. C., Iddon, B., Suschitzky, H. & Woolley, L. H. (1995). *Tetrahedron*, **51**, 10771–10794.

[bb9] Kaim, W. & Schwederski, B. (1994). In *Bioinorganic chemistry: inorganic elements in the chemistry of life, an introduction and guide, Inorganic Chemistry: A Textbook series*. Chichester: John Wiley & Sons.

[bb10] Kuş, C., Ayhan-Kilcigil, G., Can Eke, B. & Işcan, M. (2004). *Arch. Pharm. Res.* **27**, 156–163. 10.1007/BF02980099 15022715

[bb11] Li, Q. F., He, R. H., Jensen, J. O. & Bjerrum, N. J. (2003). *Chem. Mater.* **15**, 4896–4915.

[bb12] Li, W., Wang, Y., Wang, Z., Dai, L. & Wang, Y. (2011). *Catal. Lett.* **141**, 1651–1658.

[bb13] Malek, K., Puc, A., Schroeder, G., Rybachenko, V. I. & Proniewicz, L. M. (2006). *Chem. Phys.* **327**, 439–451.

[bb14] Preston, P. N. (1974). *Chem. Rev.* **74**, 279–314.

[bb15] Sheldrick, G. M. (2008). *Acta Cryst.* A**64**, 112–122. 10.1107/S0108767307043930 18156677

