# Supplementary figures and images for: Crystal structure of catena-poly[1,3-di­benzyl­benzimidazolium [[chlorido­mercurate(II)]-di-μ-chlorido]]
Source: Acta Crystallogr E Crystallogr Commun. 2015 Dec 12;71(Pt 12):m255–6. doi: 10.1107/S2056989015023427 (PMC4719861; doi:10.1107/S2056989015023427)

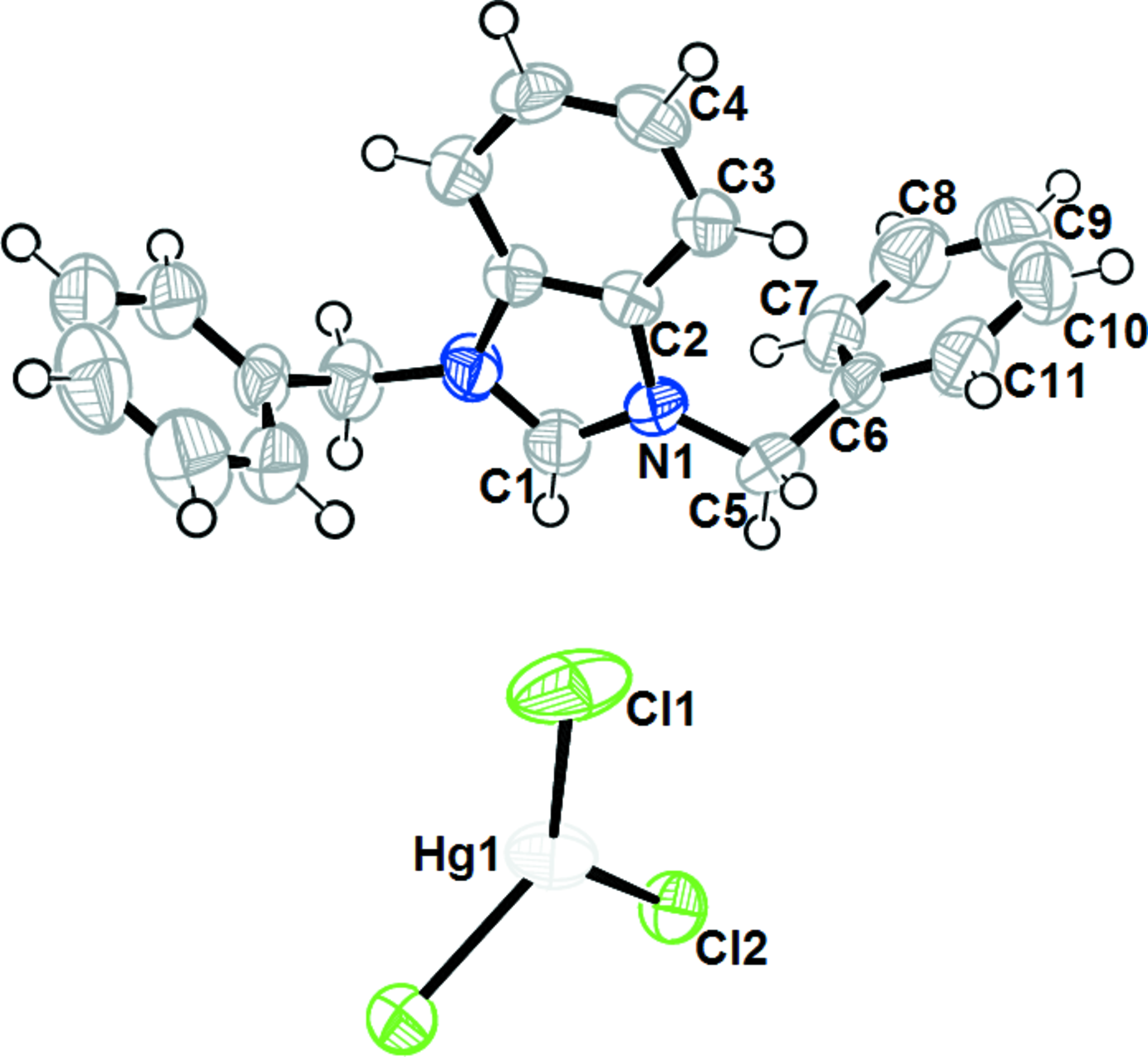

Supplement: Supplementary file 3 [file e-71-0m255-fig1.tif]

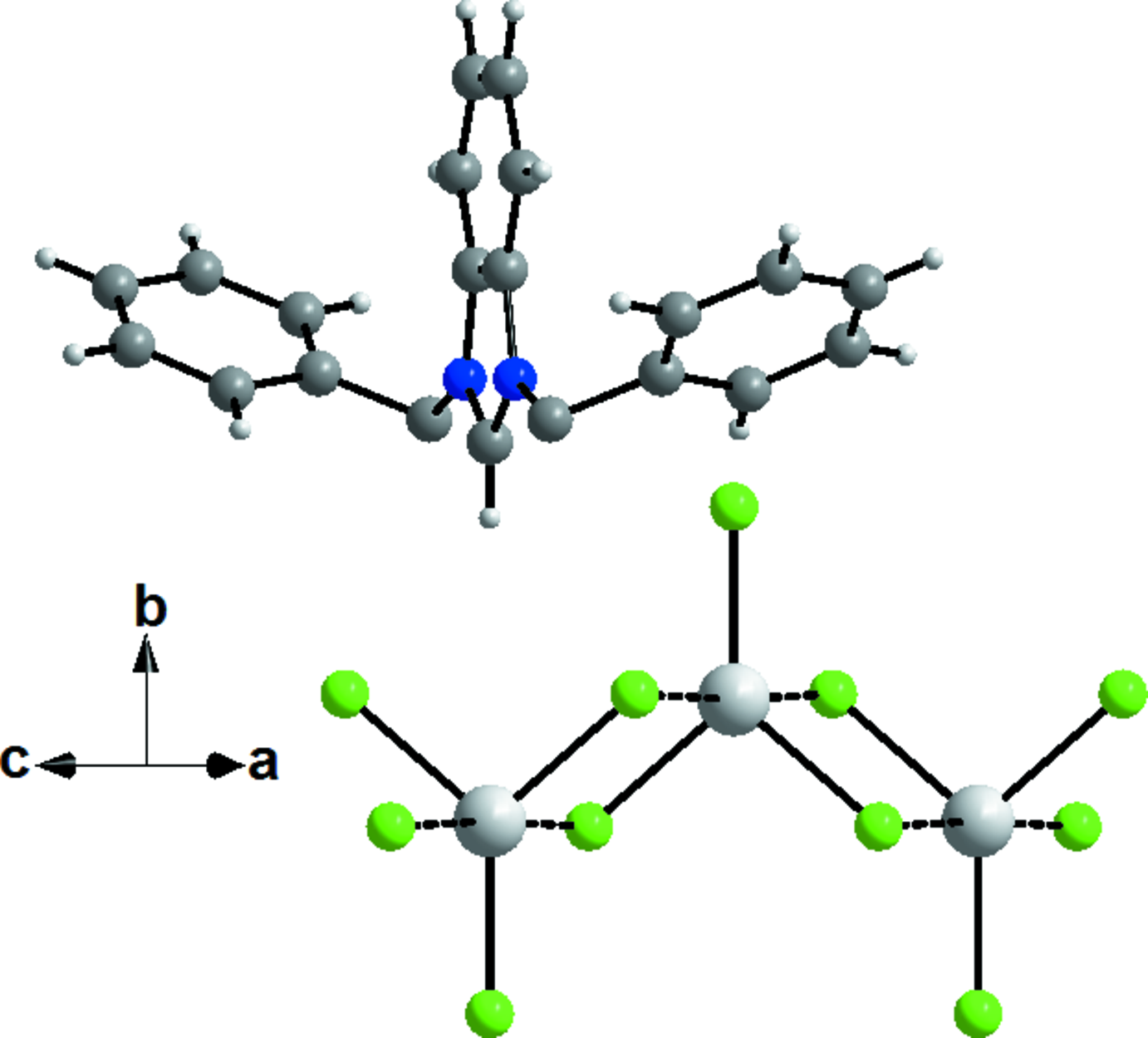

Supplement: Supplementary file 4 [file e-71-0m255-fig2.tif]

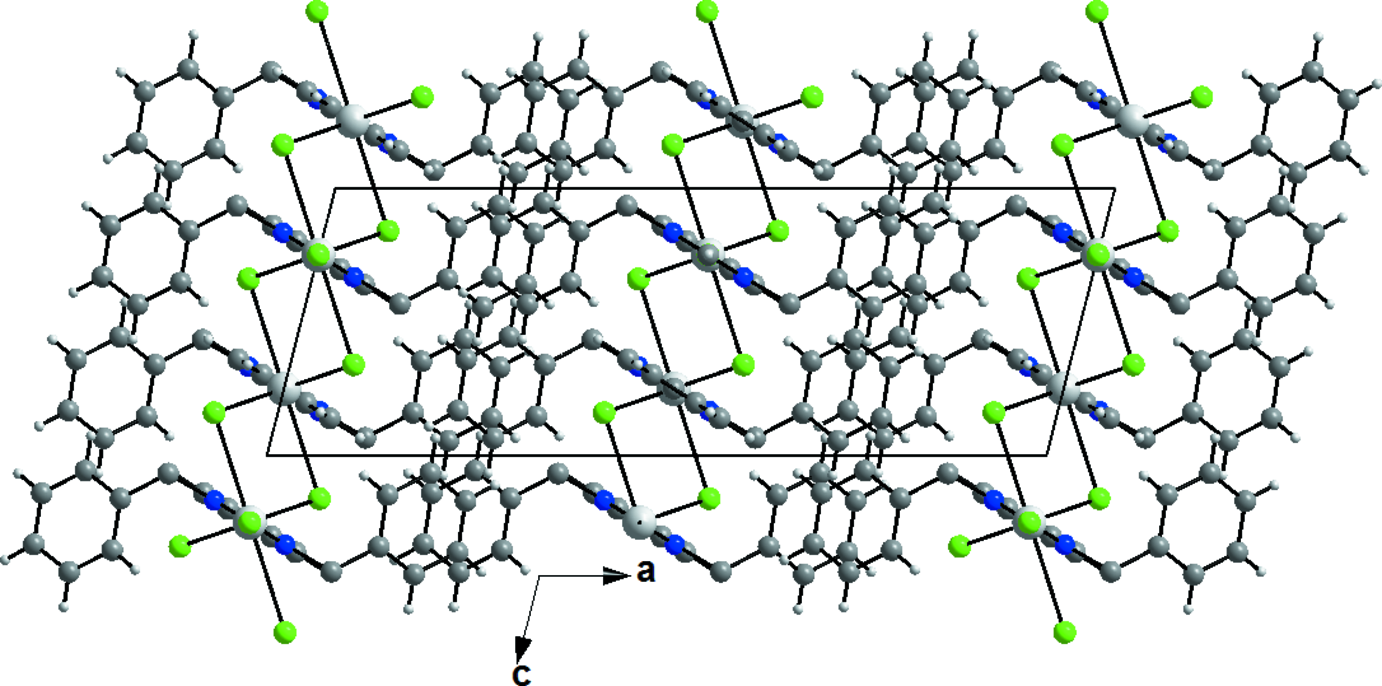

Supplement: Supplementary file 5 [file e-71-0m255-fig3.tif]

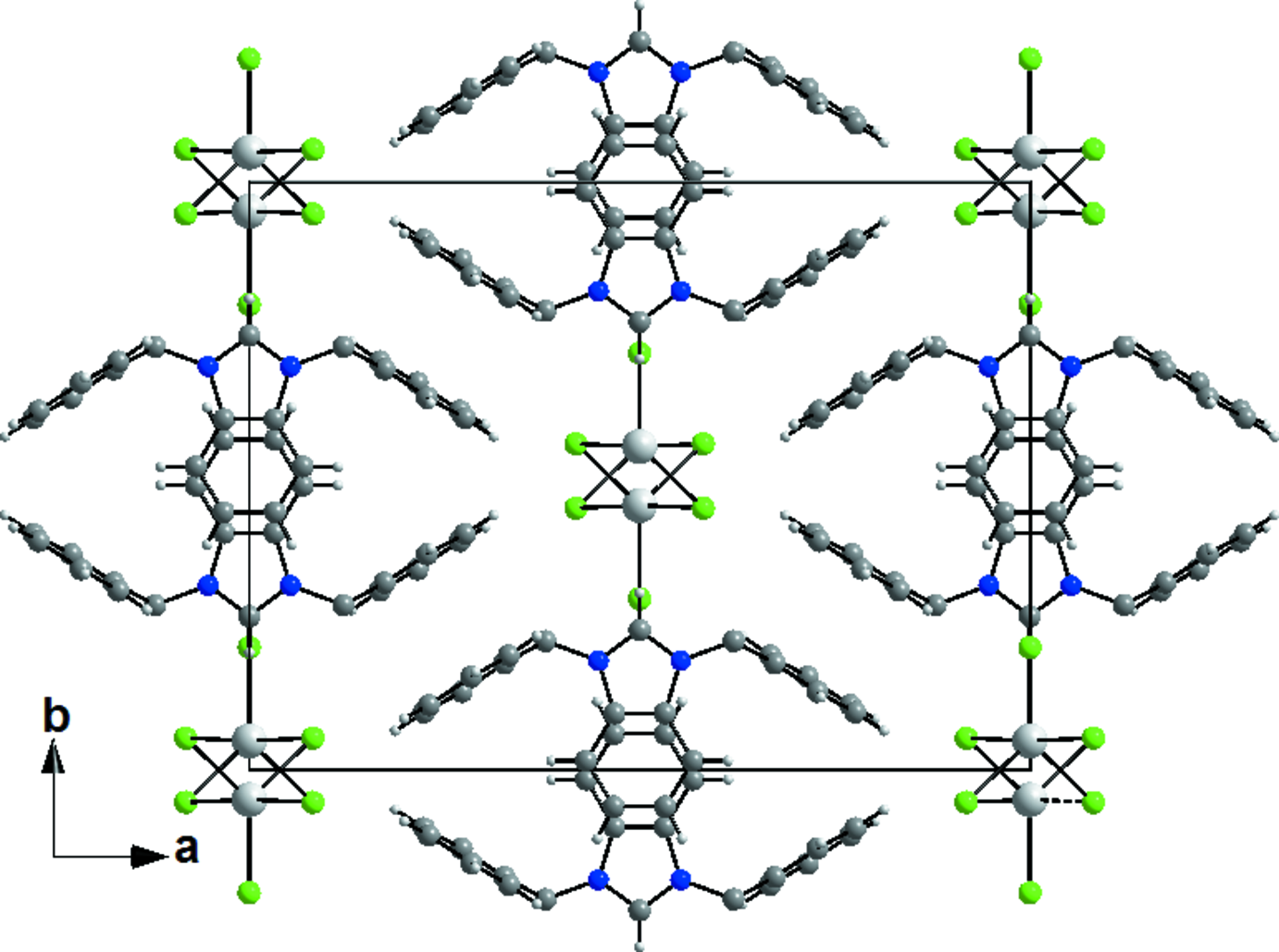

Supplement: Supplementary file 6 [file e-71-0m255-fig4.tif]
